# Association of Serum Phosphate with Low Handgrip Strength in Patients with Advanced Chronic Kidney Disease

**DOI:** 10.3390/nu13103605

**Published:** 2021-10-14

**Authors:** Ping-Huang Tsai, Hsiu-Chien Yang, Chin Lin, Chih-Chien Sung, Pauling Chu, Yu-Juei Hsu

**Affiliations:** 1 Division of Nephrology, Department of Internal Medicine, Tri-Service General Hospital, National Defense Medical Center, Taipei 114, Taiwan; tsaipinghuang@gmail.com (P.-H.T.); doc10563@mail.ndmctsgh.edu.tw (C.-C.S.); pauling.chu@gmail.com (P.C.); 2 Division of Nephrology, Department of Internal Medicine, Zuoying Branch of Kaohsiung Armed Forces General Hospital, Kaohsiung 81342, Taiwan; Ndmcofbr2@yahoo.com.tw; 3 School of Public Health, National Defense Medical Center, Taipei 114, Taiwan; xup6fup0629@gmail.com; 4 Department of Research and Development, National Defense Medical Center, Taipei 114, Taiwan; 5 Department of Biochemistry, National Defense Medical Center, Taipei 114, Taiwan

**Keywords:** chronic kidney disease, hyperphosphatemia, sarcopenia, bioimpedance, handgrip strength

## Abstract

Muscle wasting and hyperphosphatemia are becoming increasingly prevalent in patients who exhibit a progressive decline in kidney function. However, the association between serum phosphate (Pi) level and sarcopenia in advanced chronic kidney disease (CKD) patients remains unclear. We compared the serum Pi levels between advanced CKD patients with (*n* = 51) and those without sarcopenia indicators (*n* = 83). Low appendicular skeletal muscle mass index (ASMI), low handgrip strength, and low gait speed were defined per the standards of the Asian Working Group for Sarcopenia. Mean serum Pi level was significantly higher in advanced CKD patients with sarcopenia indicators than those without sarcopenia indicators (3.88 ± 0.86 vs. 3.54 ± 0.73 mg/dL; *p* = 0.016). Univariate analysis indicated that serum Pi was negatively correlated with ASMI, handgrip strength, and gait speed. Multivariable analysis revealed that serum Pi was significantly associated with handgrip strength (standardized β = −0.168; *p* = 0.022) and this association persisted even after adjustments for potential confounders. The optimal serum Pi cutoff for predicting low handgrip strength was 3.65 mg/dL, with a sensitivity of 82.1% and specificity of 56.6%. In summary, low handgrip strength is common in advanced CKD patients and serum Pi level is negatively associated with handgrip strength.

## 1. Introduction

Loss of muscle strength and muscle mass are hallmarks of sarcopenia [1]. In addition to its association with aging, sarcopenia is commonly observed in patients with chronic kidney disease (CKD) [2,3], with its prevalence increasing with CKD progression [4]. Sarcopenia is associated with poor life quality, higher risk of hospitalization, and death in populations with end-stage renal disease (ESRD) [5]. Thus, the early detection and standardized diagnosis of sarcopenia are key to preventing consecutive complications. In previous studies, the diagnosis of sarcopenia was determined solely by reduced skeletal muscle mass [6]. In 2010, the European Working Group on Sarcopenia in Older People (EWGSOP) redefined sarcopenia as a condition characterized by the presence of low muscle mass and either low muscle strength or low physical performance [7]. In 2018, the EWGSOP guidelines were revised to emphasize muscle function, and patients with low muscle strength were defined to have probable sarcopenia [1]. This revision was based on a growing body of evidence on the role of low muscle strength in predicting recurrent falls, poor healthcare outcomes, all-cause mortality, and functional decline in older adults [8,9]. This modification suggested that muscle strength is better than muscle mass in predicting adverse outcomes. Observed among the CKD population is a high prevalence (25.2%) of low handgrip strength [10], which is a predictor of composite renal outcomes defined by predialysis mortality or progression to ESRD [11].

During CKD progression, a patient’s phosphate (Pi) excretion starts to decline in stage 2 CKD and serum Pi retention becomes apparent when CKD progresses from stage 3 to stage 5. Hyperphosphatemia prevalence increases gradually to 40% in patients who have an estimated glomerular filtration (eGFR) <20 mL/min/1.73 m^2^ [12,13]. Hyperphosphatemia is proven to be associated with an increased risk of cardiovascular morbidity in patients on maintenance hemodialysis (HD) [14] and patients who have predialysis CKD [15]. Recent research has suggested that Pi toxicity can accelerate the mammalian aging process. Klotho is an antiaging protein, and mice with genetic ablation of Klotho exhibit hyperphosphatemia and premature aging phenotypes, such as short life spans, vascular calcification, osteopenia, and skeletal muscle wasting [16]. Conversely, Klotho-knockout mice that were subjected to dietary Pi restriction exhibited reduced serum Pi levels, extended life spans, and alleviated muscle wasting [17], all of which suggest an association between serum Pi levels and muscle wasting. A human study also demonstrated that a higher serum Pi level was associated with reduced vigorous physical activity independent of body mass index (BMI) and eGFR [18]. In contrast to previous rodent [16] and human studies [18,19], Mori et al. reported that, among ESRD patients who received HD treatment, the sarcopenia group exhibited significantly lower serum Pi levels compared with the nonsarcopenia group [20]. However, few studies have explored the association of serum Pi level with muscle wasting and muscle strength in patients who have advanced CKD.

In this study, we aimed to clarify the association between serum Pi and sarcopenia phenotype in patients with advanced CKD. We hypothesized that serum Pi is negatively associated with muscle strength, muscle mass, and physical performance in these patients. To test this hypothesis, we conducted a cross-sectional study involving patients with advanced CKD to explore the association of serum Pi with handgrip strength, gait speed, and appendicular skeletal muscle mass index (ASMI), which was assessed through bioelectrical impedance analysis (BIA). We discovered that serum Pi level has an independent negative correlation with handgrip strength in patients with advanced CKD.

## 2. Materials and Methods

### 2.1. Study Design and Participants

In this cross-sectional study, 134 patients at the Tri-Service General Hospital, Taipei, Taiwan, were enrolled between August 2017 and June 2018. The objective was to investigate the association of serum Pi with muscle strength and mass among patients with CKD, and they were staged based on their eGFR. Ambulatory male and female patients who had stage 3–5 CKD, were aged ≥20 years, and were receiving conservative treatment were included in the study. Patients who were undergoing chronic dialysis or had a history of chronic dialysis or renal transplantation were excluded. Furthermore, patients with the following conditions were excluded: acute illness, edema, medical implants, joint replacement, cancer, stroke, neuropathy, Parkinson’s disease, heart failure, liver disease, chronic obstructive pulmonary disease, arthritis, amputation, primary muscular disorders, and other systemic causes for muscle wasting (with limited ability to perform the tests necessary for assessing muscle mass and strength).

### 2.2. Biochemical Profiles

Automated methods (AU 5000 chemistry analyzer; Olympus, Tokyo, Japan) were used to conduct serum biochemical analyses and the Modification of Diet in Renal Disease formula was used to calculate eGFR. The SYSMEX XE-5000 cell counter (SYSMEX, Kobe, Japan) was used to analyze hemoglobin and platelet count.

### 2.3. Body Composition and BIA

Mid-upper arm circumference (MUAC) was measured at the midpoint between the tip of the shoulder and the tip of the elbow. Triceps skinfold (TSF) thickness was measured on the back of the left arm at the midpoint between the acromial process of the scapula and olecranon process of the ulna.

The InBody 720 (Biospace, Seoul, Korea) is a multifrequency impedance plethysmograph body composition analyzer; its results were validated against dual-energy X-ray absorptiometry [21]. The body composition analyzer assessed the resistance of the bilateral arms, bilateral legs, and trunk in broadband frequencies (1, 5, 50, 250, 500, and 1000 kHz) and their reactance in mean frequencies (5, 50, and 250 kHz). Segmental impedance values were then summated to obtain the total body impedance value. Per the manufacturer’s recommendation, the thumb was placed on the thumb electrode and the other four fingers were placed along the palm electrode. Participants stood on the sole electrodes and extended their arms and legs during the procedure to avoid direct contact between body segments. The participant’s body height, age, and sex were entered into the manufacturer’s software, Lookin’ Body Version 3.0 (Biospace, Seoul, Korea), before the bioelectrical impedance measurements were taken. Fat mass (FM), fat-free mass (FFM), and total body skeletal muscle mass (TSM) were calculated. The sum of the total lean body mass of the four limbs was the appendicular skeletal muscle mass (ASM), which was divided by height squared to obtain the ASMI (kg/m^2^).

### 2.4. Assessment of Sarcopenia

Handgrip strength was measured using a hydraulic dynamometer (Exacta, North Coast Medical, Arcata, CA, USA). The participants performed voluntary isometric contractions with their dominant hand while seated; their upper arm was aligned vertically and their elbow was flexed to 90°. The values of three successive trials were recorded and the mean was registered. Gait speed was measured in a 5 m walk test; the participants stood with their toes contacting the starting mark, and the time they took from the first step to the first footfall touching or crossing the finish line was recorded. For the walk test, the use of walking aids was prohibited and each participant was tested twice, with the faster time being used for the analyses.

The Asian Working Group for Sarcopenia (AWGS) 2019 criteria [22] defined the following cutoff values for the aforementioned tests: handgrip strength of <28 kg for men and <18 kg for women, gait speed of ≤1.0 m/s, and ASMI (determined through BIA) of <7.0 kg/m^2^ for men and <5.7 kg/m^2^ for women. In this study, we defined the sarcopenia phenotype as the presence of decreased grip strength, slow gait speed, or low muscle mass per the AWGS 2019 criteria. Patients who did not meet any of the criteria were assigned to the control group.

### 2.5. Statistical Analyses

Continuous and categorical variables are expressed as means ± standard deviations and numbers (percentages), respectively. Statistical analyses were carried out using SPSS for Windows version 26.0 (SPSS, Chicago, IL, USA). A *p*-value of ≤0.05 was considered statistically significant. To compare the differences between two groups of parametric variables, independent *t*-tests and chi-squared tests were performed for normally distributed and categorical variables, respectively. Univariable and multivariable linear regression analyses were conducted to examine the association of serum Pi with muscle strength, muscle mass, and gait speed. Receiver operating characteristic (ROC) curves were plotted to analyze the predictive ability of serum Pi for sarcopenia indicators and to determine an optimal cutoff point for serum Pi.

## 3. Results

### 3.1. Baseline Characteristics

A total of 134 patients with advanced CKD were enrolled. The baseline characteristics of the study population, subgroups segmented by sarcopenia phenotype, and control group are shown in Table 1. The patients’ mean age was 65.34 ± 12.96 years, and 69.4% of them were men. The mean and median serum Pi levels were 3.72 ± 0.81 mg/dL and 3.75 mg/dL, respectively. According to the AWGS 2019 criteria, sarcopenia prevalence in the study group was 6.7%. The analysis of the sarcopenia indicators revealed that 32 patients (23.9%) had low handgrip strength, 54 patients (40.3%) had low gait speed, 18 patients (13.4%) had low ASMI, and 70 patients (52.2%) exhibited at least one indicator that is representative of the sarcopenia phenotype. Compared with the control group, the patients who had CKD and the sarcopenia phenotype had significantly higher mean age and serum Pi, and significantly lower MUAC, hemoglobin, handgrip strength, gait speed, ASMI, and muscle mass surrogates.

### 3.2. Clinical Characteristics and Sarcopenia Indicators of CKD Patients Stratified by Serum Pi Tertiles

To clarify whether serum Pi level is associated with muscle strength or muscle mass in patients with CKD, we analyzed the characteristics of such patients and stratified the patients into serum Pi tertiles (Table 2). Patients in the lower serum Pi tertile tended to be male; patients in the highest serum Pi tertile had lower hemoglobin and serum total calcium levels but higher BUN, creatinine, and uric acid levels relative to the patients in the lower tertiles. However, the three tertiles did not exhibit any significant differences in serum albumin levels (Table 2).

### 3.3. Association of Serum Pi Level with Body Composition and Sarcopenia Indicators

**Figure 1:**
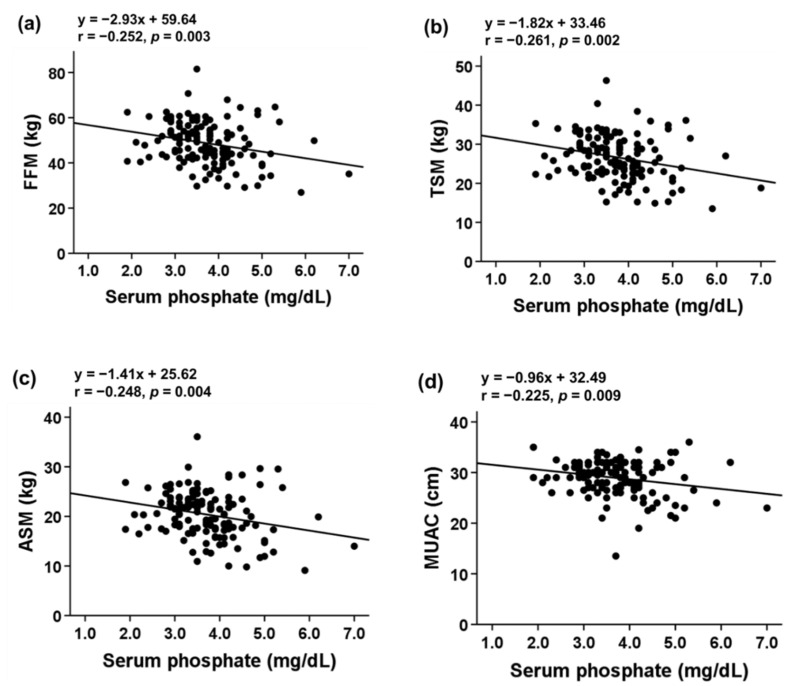

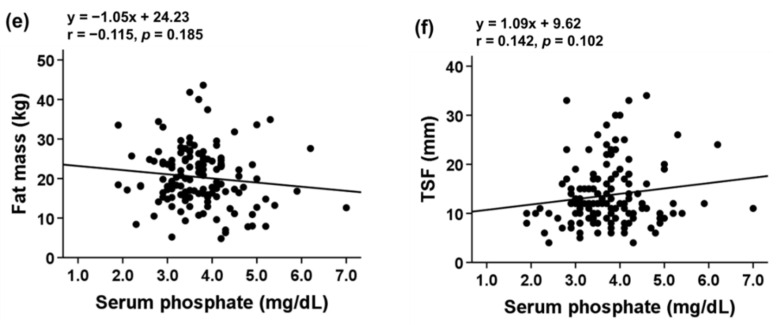
Correlation of serum phosphate with (**a**) FFM, (**b**) TSM, (**c**) ASM, (**d**) MUAC, (**e**) fat mass, and (**f**) TSF in patients with stage 3–5 CKD. Abbreviations: ASM, appendicular skeletal muscle mass; FFM, fat-free mass; MUAC, mid-upper arm circumference; TSF, triceps skinfold; TSM, total body skeletal muscle mass.

**Figure 2:**
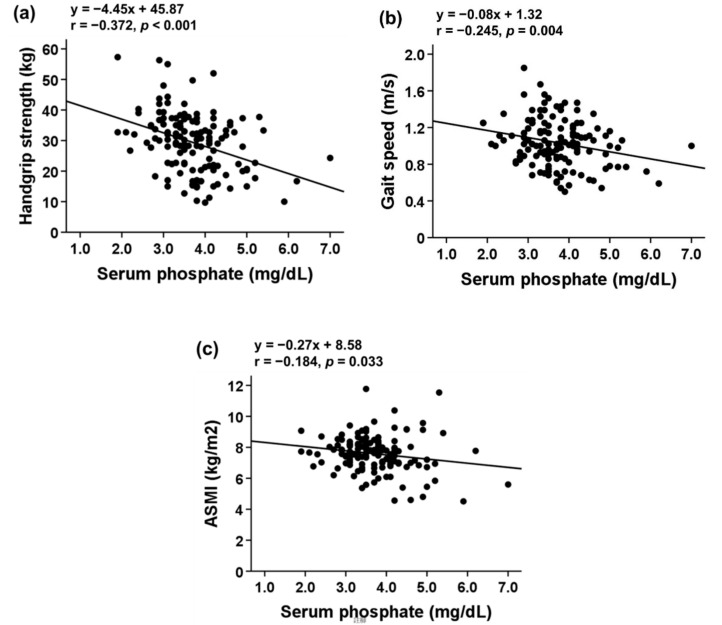
Correlation of serum phosphate with (**a**) handgrip strength (**b**) gait speed, and (**c**) ASMI in patients with stage 3–5 CKD. Abbreviations: ASM, appendicular skeletal muscle mass; FFM, fat-free mass; TSM, total body skeletal muscle mass.

Serum Pi level was inversely correlated with FFM (r = −0.252; *p* = 0.003), TSM (r = −0.261; *p* = 0.002), ASM (r = −0.248; *p* = 0.004), and MUAC (r = −0.225; *p* = 0.009). No significant relationship between serum Pi and FM (r = −0.115; *p* = 0.185) or TSF (r = 0.142; *p* = 0.102) was observed (Figure 1). Serum Pi level was inversely correlated with sarcopenia indicators (Figure 2).

To identify the independent association, the standardized β (std. β) coefficients for the association of serum Pi with handgrip strength, gait speed, and ASMI were calculated. A univariate analysis of the association of serum Pi with handgrip strength indicated a significant association with a std. β of −0.372 (*p* < 0.001; Table 3, Model 1).

Subsequently, we performed multivariable analyses with adjustments for age and sex and obtained a std. β of −0.214 (*p* = 0.001; Table 3, Model 2). Additional adjustments for eGFR resulted in a std. β of −0.216 (*p* = 0.001; Table 3, Model 3). Further adjustments for BMI and MUAC did not significantly change the results (std. β = −0.184, *p* = 0.004; Table 3, Model 4). Finally, additional adjustments for hemoglobin, uric acid, and total calcium in Model 5 (R^2^ = 0.656) did not significantly change the association of serum Pi with handgrip strength (std. β = −0.168 *p* = 0.022; Table 3).

A univariate analysis of the association of serum Pi with gait speed indicated a significant association with a std. β of −0.245 (*p* = 0.004; Table 3, Model 1). Additional adjustments for age and sex did not significantly change the results (std. β = −0.244, *p* = 0.005; Table 3, Model 2). Further adjustments for eGFR resulted in a std. β of −0.224 (*p* = 0.010; Table 3, Model 3). Adjustments for BMI and MUAC did not significantly change the results (std. β = −0.197, *p* = 0.020). However, the association of serum Pi with gait speed was not observed after additional adjustments were made for the potential confounders in Model 5 (std. β = −0.160, *p* = 0.121).

To investigate the association of serum Pi with ASMI, a crude analysis was performed and a significant association was observed (std. β = −0.184, *p* = 0.033). However, the association was not observed after adjustments were made for age and sex. Adjustments made for all potential confounders did not reveal any association between serum Pi with ASMI.

### 3.4. Determination of Optimal Serum Pi Cutoff Point for Low Handgrip Strength

**Figure 3:**
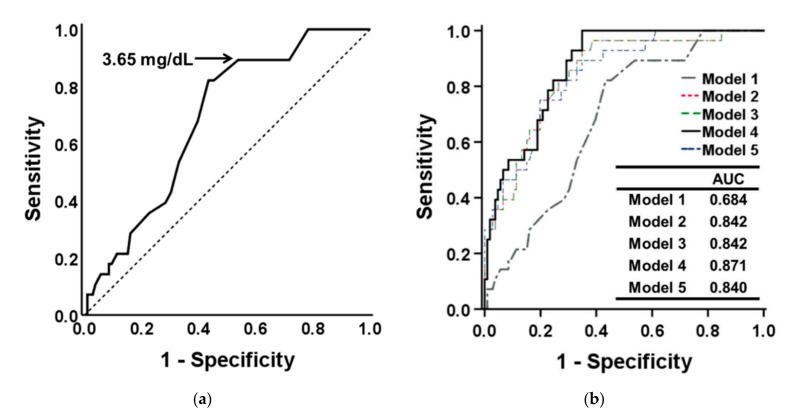
(**a**) Receiver operating characteristic (ROC) curve for determining the optimal serum Pi cutoff point to predict low handgrip strength in patients with CKD. (**b**) ROC curves for multivariate regression models designed to predict low handgrip strength in patients who have CKD. Model 1: Crude analysis. Model 2: Model 1 adjusted for age and sex. Model 3: Model 2 additionally adjusted for eGFR. Model 4: Model 3 additionally adjusted for BMI and MAC. Model 5: Model 4 additionally adjusted for hemoglobin, uric acid, and total calcium.

ROC analysis was performed to determine the optimal serum Pi cutoff point for patients with CKD and low handgrip strength (Figure 3a). An area under the curve (AUC) of 0.684 (95% CI: 0.586–0.783, *p* = 0.003) was obtained for the population, and a serum Pi cutoff of 3.65 mg/dL (82.1% sensitivity and 56.6% specificity) was proposed. Figure 3b presents the additive analysis for hierarchical adjustment and indicates that the AUCs were 0.842, 0.842, and 0.871 in Model 2, 3, and 4, respectively. Model 4, which comprised serum Pi, age, sex, eGFR, BMI, and MUAC, exhibited the highest model discrimination (AUC = 0.871, 95% CI: 0.810–0.932, *p* < 0.001; Figure 3b). However, the additional laboratory data did not aid the prediction of low handgrip strength.

## 4. Discussion

Currently, the standards for diagnosing sarcopenia in patients with advanced CKD are not well established, and the reported prevalence of sarcopenia varies from 4% to 49% depending on the cutoff value that is used [23]. To date, only a few studies have reported the prevalence of low handgrip strength in patients with advanced CKD [24,25,26]. In the present study, the prevalence of low handgrip strength was 20.9%. In Europe, the Swedish Renal Exercise trial analysis reported a low handgrip strength prevalence of 26% among patients who had advanced CKD [25], and a cross-sectional study conducted in Italy reported a higher prevalence of 63% in patients aged >65 [26]. In Asia, Lee et al. reported a low handgrip strength prevalence of 25.2% in South Korea [10], and a cross-sectional study in Taiwan indicated a low handgrip strength prevalence of 39.7% [27]. Emerging findings suggest that, compared with muscle mass, handgrip strength is more significantly correlated with clinical outcomes in older adults and ESRD patients [28,29,30,31]. Moreover, EWGSOP2 suggests that handgrip strength decreases faster than muscle mass and that it is a more sensitive screening test for sarcopenia [1]. These findings indicate that low handgrip strength is common in patients with CKD, and the early detection of low handgrip strength and identification of risk factors are key considerations in CKD care.

Older age and male sex are associated with the development of low handgrip strength [32,33]. In this study, the serum Pi level of patients with advanced CKD and the sarcopenia phenotype was significantly higher relative to the control group. Our univariate analysis revealed that serum Pi level was negatively correlated with handgrip strength, gait speed, and ASMI. Furthermore, serum Pi level had a significant negative correlation with handgrip strength after adjustment for possible confounders. For every incremental increase in serum Pi level by 1 mg/dL, handgrip strength was reduced by 1.972 kg on average. These findings indicate that serum Pi level is independently associated with low handgrip strength in patients with advanced CKD. Consistent with our observations, Peri-Okonny et al. assessed the relationship between serum Pi and physical activity in 1603 participants, and they discovered that a higher serum Pi was independently associated with increased sedentary time and less time spent engaging in moderate-to-vigorous physical activity [18]. A study that analyzed data from the National Health and Nutrition Examination Survey also revealed that, among older participants, a high serum Pi (i.e., serum Pi levels in the higher quartiles) was associated with low muscle strength [34].

In this study, we demonstrated that the serum Pi level was significantly higher in advanced CKD patients with sarcopenia phenotype than those without. To further clarify the association among serum Pi level, muscle strength, and muscle mass, we stratified these patients by serum Pi into tertiles and found that patients in the highest serum Pi tertile had lower handgrip strength relative to the patients in the lower tertiles. In line with our observations, growing evidence supports a correlation between higher serum Pi and lower muscle strength. Our present and previous in vitro studies have demonstrated that rodent muscle cells cultured in a high-Pi-containing medium exhibit muscle atrophy characterized by the accumulation of myostatin, accelerated senescence with integrin-linked kinase overexpression, reduced proliferative capacity, activation of autophagy, reductions in mitochondrial membrane potential, and activation of Nrf2 signaling [35,36,37,38]. Furthermore, increased Pi can reduce Ca^2+^ release from the sarcoplasmic reticulum, which may lead to reduced cross-bridge activity and impaired muscle contraction [39,40]. Similarly to the aforementioned in vitro findings, an animal study revealed that mice subjected to a high-Pi diet developed exercise intolerance, which was determined by conducting a treadmill exercise test and assessing altered fatty acid metabolism in mice skeletal muscles [18]. The pathogenic role of high Pi in muscle wasting was further supported by a human study, which demonstrated that knee extensor fatigability was greater in older adults relative to younger adults and revealed decreased pH and increased Pi levels (which were determined using phosphorus nuclear magnetic resonance spectroscopy) in the muscles of older adults [41].

Hyperphosphatemia is associated with an increased risk of cardiovascular morbidity and mortality in patients on maintenance HD [42]; it is also associated with cardiovascular risk in patients who have CKD but are not on dialysis [43]. In patients who have moderate CKD with a mean eGFR of 50.6 mL/min per 1.73 m^2^, higher Pi levels are associated with an increased prevalence of vascular calcification that is independent of serum parathyroid hormone (PTH) and calcitriol levels [15,44]. The Kidney Disease: Improving Global Outcomes (KDIGO) workgroup suggested that the elevated Pi levels of patients with CKD G3a–G5D ought to be reduced to normal levels [45]. In the present study, the optimal serum Pi cutoff value for predicting low handgrip strength was 3.65 mg/dL (AUC = 0.684, 95% CI: 0.586–0.783). Adjusting the prediction model for age, sex, and eGFR resulted in the AUC increasing from 0.684 to 0.871. Our findings support the position that the serum Pi of patients with advanced CKD should be maintained within a normal range; this position is consistent with KDIGO guidelines.

The present study has limitations. Firstly, we did not conduct a longitudinal survey and, consequently, could not verify any causative relationship between serum Pi and handgrip strength. Secondly, the dietary records of Pi intake, which could be a major confounder, were unavailable. Thirdly, although we excluded patients who had CKD and peripheral edema concurrently, BIA measurements could still be influenced by the hydration status of participants. Fourthly, we did not measure the participants’ serum PTH levels, which are proven to be a muscle-wasting factor. Fifthly, the specificity of serum Pi in predicting low handgrip strength was not so high. Thus, consistent with the recommendations in AWGS 2019 criteria, measuring handgrip strength rather than measuring serum Pi is still the useful screening test for sarcopenia in patients with advanced CKD, especially in those with hyperphosphatemia.

## 5. Conclusions

In conclusion, the present study demonstrates that a high serum Pi concentration is significantly associated with low handgrip strength in patients with advanced CKD. Moreover, serum Pi may be used to predict low handgrip strength. A large cohort study is warranted to verify the causal relationship between serum Pi and handgrip strength and to investigate the optimal serum Pi concentration range for preventing low handgrip strength in patients who have advanced CKD.

## Figures and Tables

**Table 1:** Baseline characteristics of patients with stage 3–5 CKD and comparison of patients with sarcopenia phenotype and controls.

Parameters	Total (*n* = 134)	Sarcopenia Phenotype (*n* = 70)	Control (*n* = 64)	*p* Value
Age (years)	65.34 ± 12.96	68.87± 12.19	61.48 ± 12.75	0.001
Male sex, *n* (%)	93 (69.4)	40 (57.1)	53 (82.8)	0.001
Height (cm)	162.83 ± 8.93	160.54 ± 10.02	165.33 ± 6.79	0.002
Weight (kg)	68.13 ± 13.23	64.87 ± 14.47	71.70 ± 10.74	0.003
BMI (kg/m^2^)	25.60 ± 4.11	25.06 ± 4.66	26.19 ± 3.33	0.114
MUAC (cm)	28.93 ± 3.46	27.74 ± 3.67	30.23 ± 2.69	<0.001
TSF (mm)	13.68 ± 6.26	13.67 ± 5.63	13.69 ± 6.93	0.988
eGFR	35.33 ± 16.61	34.18 ± 16.29	36.59 ± 17.00	0.404
Diabetes mellitus, *n* (%)	63 (47.0)	34 (48.6)	29 (45.3)	0.706
Hypertension, *n* (%)	99 (73.9)	54 (77.1)	45 (70.3)	0.369
Coronary artery disease, *n* (%)	31 (23.1)	17 (24.3)	14 (21.9)	0.741
Hyperlipidemia, *n* (%)	91 (67.9)	44 (62.9)	47 (73.4)	0.190
Laboratory measurements				
Hemoglobin (g/dL)	12.37 ± 2.02	11.99 ± 1.93	12.79 ± 2.05	0.030
Platelet count (×10^9^/L)	221.31 ± 58.51	214.31 ± 64.53	227.05 ± 53.25	0.365
Total cholesterol (mg/dL)	166.96 ± 37.73	162.72 ± 33.90	171.36 ± 41.17	0.227
HDL cholesterol (mg/dL)	46.28 ± 18.92	46.00 ± 21.38	46.50 ± 17.14	0.557
Triglyceride (mg/dL)	161.98 ± 118.42	168.66 ± 139.64	155.19 ± 92.71	0.524
Blood urea nitrogen (mg/dL)	36.25 ± 19.90	39.67 ± 22.39	32.58 ± 16.25	0.053
Creatinine (mg/dL)	2.56 ± 1.83	2.79 ± 2.17	2.30 ± 1.34	0.123
Uric acid (mg/dL)	6.10 ± 1.71	6.28 ± 1.72	5.90 ± 1.69	0.197
Total calcium (mg/dL)	9.33 ± 0.54	9.33 ± 0.57	9.33 ± 0.51	0.959
Glucose (mg/dL)	110.48 ± 31.24	116.22 ± 37.17	105.22 ± 23.72	0.059
Alanine aminotransferase (U/L)	22.00 ± 13.75	22.17 ± 13.69	21.85 ± 13.92	0.907
Albumin (g/dL)	4.16 ± 0.41	4.11 ± 0.40	4.21 ± 0.41	0.191
Phosphate (mg/dL)	3.72 ± 0.81	3.88 ± 0.86	3.54 ± 0.73	0.016
Bioelectrical impedance analysis				
FM%	29.01 ± 7.83	29.74 ± 8.92	28.22 ± 6.40	0.260
FM (kg)	20.32 ± 7.41	19.83 ± 8.12	20.86 ± 6.56	0.424
FFM (kg)	48.73 ± 9.49	45.67 ± 10.42	52.07 ± 7.04	<0.001
TSM (kg)	26.69 ± 5.67	24.75 ± 6.11	28.81 ± 4.26	<0.001
ASM (kg)	20.36 ± 4.64	18.97 ± 5.22	21.88 ± 3.33	<0.001
Sarcopenia indicator				
Handgrip strength (kg)	29.33 ± 9.73	24.01 ± 7.96	35.15 ± 8.04	<0.001
Gait speed (m/s)	1.03 ± 0.26	0.87 ± 0.20	1.21 ± 0.19	<0.001
ASMI (kg/m^2^)	7.59 ± 1.18	7.23 ± 1.34	7.97 ± 0.84	<0.001

Abbreviations: ASM, appendicular skeletal muscle mass; ASMI, appendicular skeletal muscle mass index; BMI, body mass index; eGFR, estimated glomerular filtration rate; FM, fat mass; FFM, fat-free mass; FM%, fat mass percentage; HDL, high-density lipoprotein; MUAC, mid-upper arm circumference; MM, muscle mass; TSF, triceps skinfold; TSM, total body skeletal muscle mass.

**Table 2:** Participants stratified by serum phosphate tertiles.

Parameters	Serum Phosphate Tertiles			*p*
	Lower (*n* = 43)	Middle (*n* = 47)	Upper (*n* = 44)	
Age (years)	66.98 ± 10.76	64.36 ± 14.38	64.80 ± 13.45	0.600
Male sex, *n* (%)	39 (90.7)	32 (68.1)	22 (50.0)	<0.001
Height (cm)	166.79 ± 6.82	162.28 ± 9.02	159.55 ± 9.33	0.001
Weight (kg)	71.05 ± 10.60	70.19 ± 12.42	63.07 ± 15.07	0.007
BMI (kg/m^2^)	25.46 ± 2.78	26.67 ± 4.30	24.59 ± 4.74	0.051
MUAC (cm)	29.78 ± 2.22	29.19 ± 3.61	27.83 ± 4.04	0.025
TSF (mm)	12.14 ± 5.26	14.62 ± 6.03	14.18 ± 7.18	0.139
eGFR	39.76 ± 14.89	34.35 ± 16.85	32.05 ± 17.36	0.084
Diabetes mellitus, *n* (%)	17 (39.5)	24 (51.1)	22 (50.0)	0.489
Hypertension, *n* (%)	28 (65.1)	34 (72.3)	37 (84.1)	0.126
Coronary artery disease, *n* (%)	9 (20.9)	8 (17.0)	14 (31.8)	0.226
Hyperlipidemia, *n* (%)	30 (69.8)	33 (70.2)	28 (63.6)	0.759
Laboratory tests				
Hemoglobin (g/dL)	13.63 ± 1.89	12.44 ± 1.75	11.19 ± 1.72	<0.001
Platelet count (×10^9^/L)	214.73 ± 59.90	213.35 ± 44.60	236.61 ± 69.61	0.456
Total cholesterol (mg/dL)	165.16 ± 35.03	174.07 ± 40.99	160.97 ± 35.97	0.287
HDL Cholesterol (mg/dL)	43.12 ± 14.42	43.10 ± 13.17	55.31 ± 28.11	0.461
Triglyceride (mg/dL)	149.80 ± 121.16	188.27 ± 126.18	146.31 ± 104.59	0.189
Blood urea nitrogen (mg/dL)	22.89 ± 7.14	31.15 ± 14.03	53.83 ± 20.63	<0.001
Creatinine (mg/dL)	1.69 ± 0.74	1.92 ± 0.84	4.07 ± 2.34	<0.001
Uric acid (mg/dL)	5.89 ± 1.60	5.79 ± 1.72	6.63 ± 1.72	0.043
Total calcium (mg/dL)	9.37 ± 0.52	9.46 ± 0.39	9.15 ± 0.64	0.019
Glucose (mg/dL)	104.56 ± 18.10	118.62 ± 39.42	108.14 ± 31.46	0.119
Alanine aminotransferase (U/L)	23.05 ± 13.53	23.47 ± 15.49	19.00 ± 11.86	0.377
Albumin (g/dL)	4.21 ± 0.33	4.22 ± 0.31	4.04 ± 0.53	0.077
Bioelectrical impedance analysis				
FM%	27.88 ± 6.87	30.67 ± 8.26	28.35 ± 8.11	0.190
FM (kg)	20.22 ± 6.39	22.12 ± 7.87	18.50 ± 7.54	0.065
FFM (kg)	51.50 ± 7.49	49.38 ± 9.47	45.33 ± 10.38	0.008
TSM (kg)	28.41 ± 4.49	27.09 ± 5.67	24.58 ± 6.12	0.005
ASM (kg)	21.66 ± 3.41	20.57 ± 4.52	18.87 ± 5.41	0.017
Sarcopenia indicator				
Handgrip strength (kg)	34.13 ± 9.45	28.18 ± 8.97	25.86 ± 9.09	<0.001
Gait speed (m/s)	1.11 ± 0.24	1.01 ± 0.28	0.98 ± 0.23	0.042
ASMI (kg/m^2^)	7.74 ± 0.75	7.73 ± 1.13	7.28 ± 1.51	0.120

Note: Categorical variables are expressed as percentages; continuous variables are expressed as means ± standard deviations. Abbreviations: ASM, appendicular skeletal muscle mass; ASMI, appendicular skeletal muscle mass index; BMI, body mass index; eGFR, estimated glomerular filtration rate; FM, fat mass; FFM, fat-free mass; FM%, fat mass percentage; HDL, high-density lipoprotein; MUAC, mid-upper arm circumference; MM, muscle mass; TSF, triceps skinfold; TSM, total body skeletal muscle mass.

**Table 3:** Association of serum phosphate level with muscle strength and mass.

	Std. β	Unstd. β (95% CI)	*p* Value
Handgrip strength			
Model 1	−0.372	−4.446 (−6.357, −2.536)	<0.001
Model 2	−0.214	−2.559 (−4.087, −1.031)	0.001
Model 3	−0.216	−2.583 (−4.136, −1.031)	0.001
Model 4	−0.184	−2.197 (−3.698, −0.696)	0.004
Model 5	−0.168	−1.972 (−3.659, −0.285)	0.022
Gait speed			
Model 1	−0.245	−0.078 (−0.131, −0.025)	0.004
Model 2	−0.244	−0.077 (−0.130, −0.024)	0.005
Model 3	−0.224	−0.071 (−0.124, −0.017)	0.010
Model 4	−0.197	−0.062 (−0.115, −0.010)	0.020
Model 5	−0.160	−0.050 (−0.113, 0.013)	0.121
ASMI			
Model 1	−0.184	−0.268 (−0.514, −0.022)	0.033
Model 2	0.008	0.011 (−0.211, 0.234)	0.921
Model 3	0.017	0.025 (−0.201, 0.250)	0.829
Model 4	0.077	0.111 (−0.032, 0.254)	0.126
Model 5	0.001	0.002 (−0.158, 0.162)	0.981

Model 1: Crude analysis. Model 2: Model 1 adjusted for age and sex. Model 3: Model 2 additionally adjusted for eGFR. Model 4: Model 3 additionally adjusted for BMI and MUAC. Model 5: Model 4 additionally adjusted for hemoglobin, uric acid, and total calcium.

## Data Availability

Data will be available upon reasonable request to the corresponding author.
